# Identifying self-reported health-related problems in home-based rehabilitation of older patients after hip replacement in China: a machine learning study based on Omaha system theory

**DOI:** 10.1186/s12911-023-02353-7

**Published:** 2023-11-21

**Authors:** Jing Chen, Fan He, Qian Wu, Li Wang, Xiaoxia Zhu, Yan Qi, JiaLing Wu, Yan Shi

**Affiliations:** 1 College of Design and Innovation, Tongji University, Shanghai, China; 2 Smart Engineering Research Institute, Shanghai Investigation, Design & Research Institute Co.,Ltd, Shanghai, China; 3 Shanghai Tenth People’s Hospital, School of Medicine, Tongji University, Shanghai, China; 4 Orthopaedics Department, Changhai Hospital, The Second Military Medical University, Shanghai, China; 5 School of Medicine, Jinggangshan University, Ji An, China; 6 School of nursing and health management, Shanghai University of Medicine & Health Sciences, Shanghai, China

**Keywords:** Hip replacement, Home-based rehabilitation, Machine learning, Older individual, Self-reported outcome

## Abstract

**Background:**

With the aging of the population, the number of total hip replacement surgeries is increasing globally. Hip replacement has undergone revolutionary advancements in surgical methods and materials. Due to the short length of hospitalization, rehabilitation care is mainly home-based. The needs and concerns about such home-based rehabilitation are constantly changing, requiring continuous attention.

**Objective:**

To explore effective methods for comprehensively identifying older patients’ self-reported outcomes after home-based rehabilitation for hip replacement, in order to develop appropriate intervention strategies for patient rehabilitation care in the future.

**Methods:**

This study constructed a corpus of patients’ self-reported rehabilitation care problems after hip replacement, based on the Omaha classification system. This study used the Python development language and implemented artificial intelligence to match the corpus data on the cooperation platform, to identify the main health-related problems reported by the patients, and to perform statistical analyses.

**Results:**

Most patients had physical health-related problems. More than 80% of these problems were related to neuromusculoskeletal function, interpersonal relationships, pain, health care supervision, physical activity, vision, nutrition, and residential environment. The most common period in which patients’ self-reported problems arose was 6 months post-surgery. The relevant labels that were moderately related to these problems were: Physiology-Speech and Language and Physiology-Mind (r = 0.45), Health-Related Behaviors-Nutrition and Health-Related Behaviors-Compliance with Doctors’ Prescription (r = 0.40).

**Conclusion:**

Physiological issues remain the main health-related issues for home-based rehabilitation after hip replacement in older patients. Precision care has become an important principle of rehabilitation care. This study used a machine learning method to obtain the largest quantitative network data possible. The artificial intelligence capture was fully automated, which greatly improved efficiency, as compared to manual data entering.

**Supplementary Information:**

The online version contains supplementary material available at 10.1186/s12911-023-02353-7.

## Summary table

**What was already known on the topic:**

As the population ages, total hip replacement surgeries are increasingly performed. Due to the short length of hospitalization, rehabilitation care is mainly home-based. The needs and concerns about such home-based rehabilitation are constantly changing, and should be continuously evaluated.

**What this study added to our knowledge:**

The corpus of patients’ self-reported rehabilitation care problems after hip replacement constructed based on the Omaha system in this study will provide a reference for the unified identification of issues related to hip replacement. The methods provided in this study provide a reference for future assessments of self-reported outcomes and intervention needs of patients. In a medical environment with limiting conditions, the mining of online clinical medical data can provide new ideas and evidence supporting precision care.

**Supplementary Information:**

The online version contains supplementary material available at 10.1186/s12911-023-02353-7.

## Introduction

In terms of population aging, China is in the rapid development stage of aging, with concomitant medical problems [1]. In older individuals, the reduction in total bone mass facilitates femoral fractures [2]. According to 5-year (2008–2012) statistics from Beijing Jishuitan Hospital [3], there is an average annual increase of 7.3% in patients older than 50 years with femoral neck fracture in China. Hip replacement surgery is the most commonly used treatment for this condition, and effectively relieves pain, improves limb function, and enhances the patient’s quality of life, but postoperative rehabilitation influences the long-term outcomes. Due to the short length of hospital stay of such patients, rehabilitation is mainly home-based [4].

In recent years, hip replacement has undergone advances in surgical methods and materials, and the focus of care has widened. The needs and concerns about patients’ home-based rehabilitation are also continuously changing. Thus, it is crucial to identify and pay attention to issues that have gone unattended in a timely and continuous manner, to consider whether the content of rehabilitation interventions currently provided is sufficient, and to make careful efforts to perform appropriate interventions from the patient’s perspective.

The current methods for surveying home-based rehabilitation patients, particularly for long-term follow-up, are often manpower- and material resource-intensive. If the patient experiences a smooth recovery post-surgery, subsequent follow-ups may be perceived as burdensome or even unnecessary [5]. Therefore, it is vital to evaluate the advantages and disadvantages of rehabilitation while contemplating improved methods for follow-up and data collection. With the increase in big data, scientific research methods can overcome various obstacles to the assessment of home-care. Innovative approaches can more efficiently identify the primary challenges and needs in home-based rehabilitation, utilizing patients’ self-reported outcomes [6]. For instance, the content published on Twitter and other social media by users with related diseases have been studied to explore the emotional experience and needs of patients in the real world [7], and could also be applied to home-based rehabilitation research. Given this context, the present study sought to use machine learning(ML) to perform data mining and analysis using such network resources.

Machine learning aids in extracting information, such as keywords and their frequency of occurrence from online text, thereby identifying individuals’ concerns [8]. This facilitates easier data collection from homebound patients, especially amidst an epidemic. On the other hand, the self-reported language of patients reflects their top health concerns. Thus, we employed a method based on supervised machine learning to extract keywords from the text through a segmentation tool [9]. However, some specific keywords can’t be identified via the existing tool of machine learning in the home rehabilitation scenario. To solve this problem, we built a third-party lexicon regarding patients’ self-reported rehabilitation care problems. The lexicon can also be applied to machine learning in other relevant scenarios. Furthermore, it can be iteratively refined in the future, and the methodology of this study presents a novel approach to home care surveys.

## Methods

**Fig. 1:**
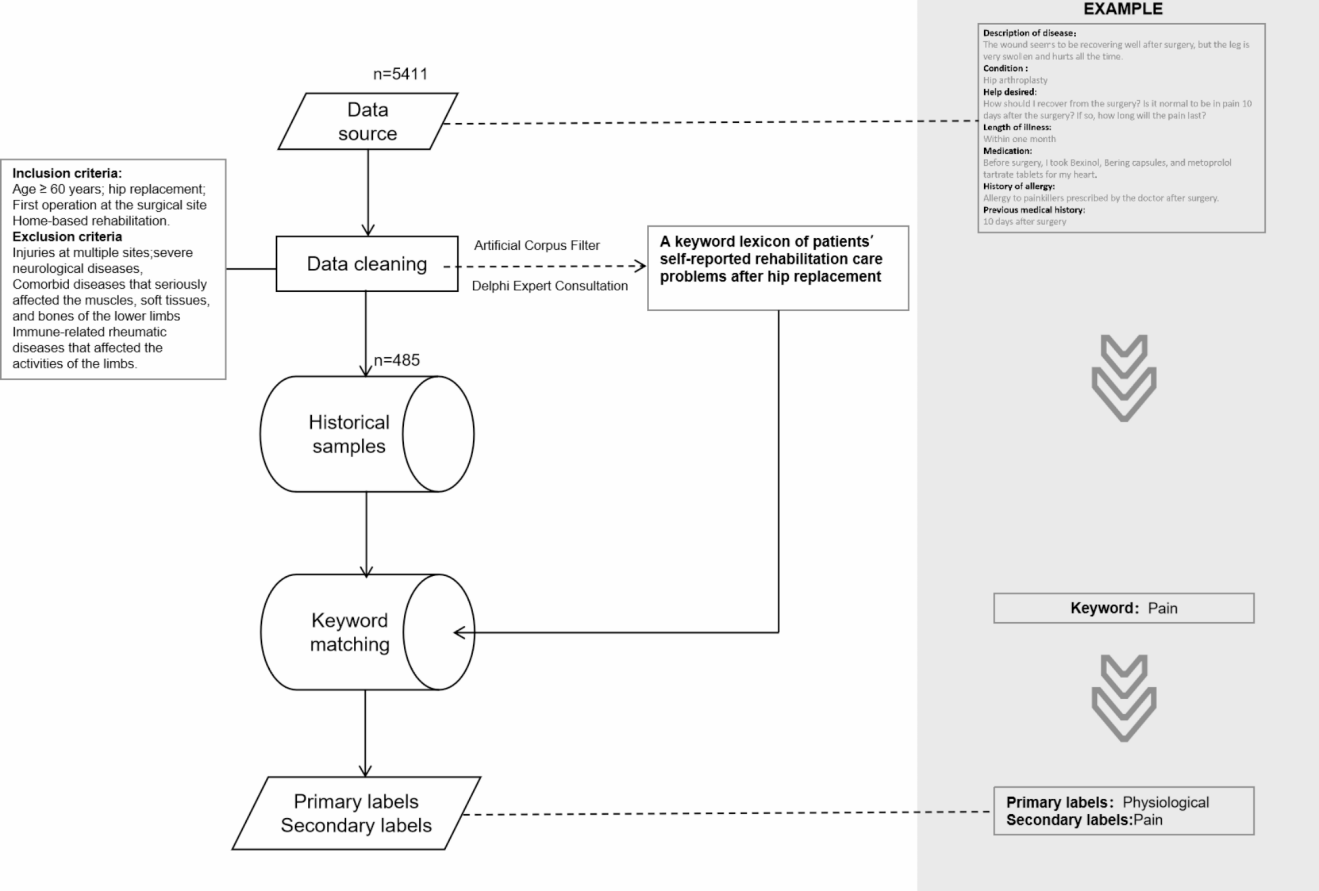
Machine learning process

We captured all data from the platform for all orthopedics departments relating to the keyword of hip replacement and obtained a total of 5411 medical records. The data was structured around 14 distinct tags, with this study particularly concentrating on the ‘help desired’ tag. After considering the inclusion and exclusion criteria, only 485 historical samples were retained in the study. At the same time, our expert team manually identified 500 pieces of corpus data from the samples, extracted keywords relevant to this study, and built a third-party lexicon about patients’ self-reporting problems based on Omaha system.It is an effective tool for collecting, sorting, recording, and analyzing patient data in clinical work [10–12]. The lexicon consists of 4 primary labels and 39 secondary labels (Fig. 1).

And then we statistically analyzed the sample set based on the lexicon, using supervised machine learning, to identify and rank the main problems patients experienced, and to analyze the correlations between problems.

### Data source

After obtaining permission from the platform, we selected all information on patients’ online consultation with orthopedic doctors on the Good Doctor Online platform that cooperated with our hospital, the platform also had relationships with several other hospitals in China in addition to us. It is a communication platform for doctors and patients, patients can describe their basic condition and main concerns anonymously, and the statements made by doctors based on patients’ self-reports are for information purposes only. All patients who ask questions are registered users of the platform and have signed an online service agreement with the platform, agreeing to the use and display of user information on the platform without compromising privacy. The study assumes the same responsibility to protect user privacy as the consultation platform and analyses the database without disclosing individual user’s private information.

The original data we received were divided into 14 tags as follows: (1) The URL, title, and content of each consultation content were extracted. (2) The content was further broken up into fine-grained information pieces according to 12 tags: disease, examination data, imaging data, description of the condition, medication status, length of illness, hospital and department visited, previous medical history, examination report, pregnancy status, history of allergies, and the help desired, with the latter being used to mark patient expressions that explicitly seek help or advice. In line with the aims of this study, the research removed some of the tags and focused on keyword extraction and analysis of the text content related to ‘help desired’. We employed a crawler program, developed with Python 3.6 and based on the Scrapy crawler framework, to automatically scrape and extract patient self-report data from online platforms.

This study collected data from the platform from April 2019 to October 2019, yielding a total sample of 5,411 cases. An anonymous case is used to explain the data source as Fig. 1 shows.

### Construction of a keyword lexicon of patients’ self-reported rehabilitation care problems after hip replacement

The patients’ self-reported language expressions were inconsistent and colloquial, and invalid corpus data, such as conjunctions and modal auxiliary words, would interfere with frequency analysis. Therefore, we initially developed a third-party lexicon, specifically designed to align with the patients’ corpus data, facilitating a structured classification and analysis through machine learning. This lexicon, based on the Omaha system, comprises 4 primary labels and 39 secondary labels, each meticulously curated to categorize and tag patient self-reports, thereby enabling a systematic extraction and analysis of relevant keywords and expressions from the patient communication data.

#### Setting primary and secondary labels

**Fig. 2:**
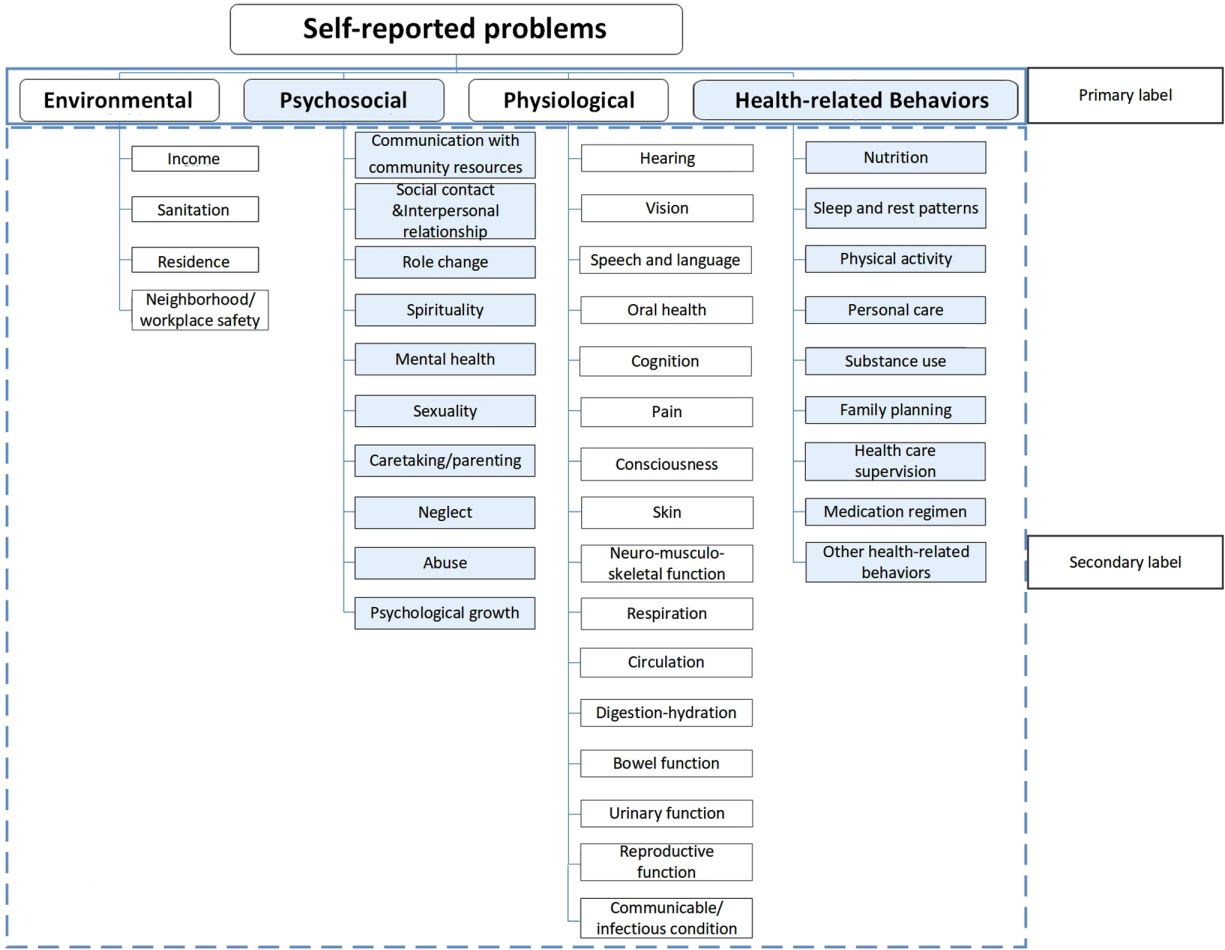
Classification criteria for self-reported problems in rehabilitation care after hip replacement in older patients, based on the Omaha classification system [10]

According to a literature review, the results of previous qualitative interviews and the preliminary artificial corpus filter results, the classification of the patients’ self-reported key problems fit well with the Omaha subsystem. Additionally, this study consolidated “sadness” in social psychology into mental health, changed “growth and development” to psychological growth, removed pregnancy and postpartum from physiological problems, and added other health-related behaviors to health-related behavior problems. After adjustments of the classifications, the criteria for classification of problems related to rehabilitation care after hip replacement in older individuals based on the Omaha system were established as four primary labels: environmental, psychosocial, physiological, and health-related behaviors, and 39 secondary labels (Fig. 2).

#### Manual recognition and expert review classification

Manual reading as well as classification and boxing were completed in April 2019 independently by the author and another orthopedic nurse with more than 10 years of experience with the 500 pieces of corpus data that had been extracted. Disagreements were adjudicated by a third person. In the preliminary filter, 332 patient keywords were identified and extracted, and were then classified and boxed according to the established primary and secondary labels using the Delphi method.

Twelve experts participated in this study. Ten experts met the following conditions: (1) Experts in medicine, nursing, or psychology engaged in bone and joint research, with rich scientific research experience, who had presided over or conducted research in related fields, and had a master’s degree or above. (2) Medical staff engaged in clinical practice involving bone and joints, with more than 5 years of medical care or nursing experience, and with a bachelor’s degree or above. The remaining two were computer and statistics experts, and provided guidance on the feasibility of corpus and data analysis. The mean self-evaluation score of the 10 experts for familiarity with the survey content was 0.87, the judgment coefficient was 0.90, the comprehensive authority coefficient obtained was 0.89. The coordination coefficient for expert opinions in the two rounds of consultation was 0.427 and 0.413, respectively, which was statistically significantly different by chi-square test.

### Keyword matching with supervised learning

For supervised machine learning [13], natural language was processed, and machine processing was used to recognize human natural language. Supervised learning was used to match keywords with corresponding labels based on the constructed lexicon.

#### Pre-processing and filtering/data cleaning

As our study focused on problems of home-based rehabilitation care after hip replacement in older patients we filtered related health problems from the massive data. The conditional filter inclusion criteria, prior to matching, were as follows: age ≥ 60 years; hip replacement; first operation at the surgical site; and home-based rehabilitation. The exclusion criteria included: injuries at multiple sites; severe neurological diseases, such as stroke, affecting limb activities; comorbid diseases that seriously impacted the muscles, soft tissues, and bones of the lower limbs; or immune-related rheumatic diseases affecting limb activities.

The number of historical samples after filtering according to the above inclusion and exclusion criteria was 485.

#### Keyword matching

**Fig. 3:**
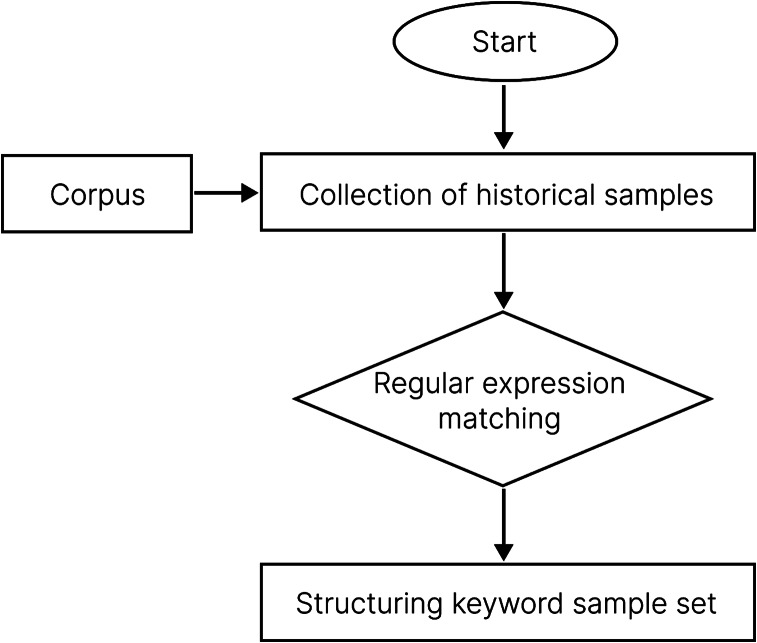
Structured data rearrangement model flowchart [14]

The regular matching method was used for batch processing of historical samples. Regular expressions can be used to determine whether a given string conformed to the filtering logic of regular expression (called “matching”) [14], to obtain the required information from a string. Keywords that matched the constructed lexicon were extracted from the patients’ self-reported language, providing a structured keyword sample set.We used the process shown in Fig. 3 to complete the keyword matching.

#### Calculation of frequency of labels corresponding to keywords in patients’ self-reports

Statistical analysis was conducted on the patients’ health problems, and the frequency and ranking of primary and secondary labels in the matching labels determined. If self-reported keywords from the same patient appeared several times in the same level of a label dimension, this secondary label was counted as appearing once for this patient. For example, if a patient reported low income (keyword appearing for the first time) and low salary (keyword appearing for the second time), the algorithm determined that the patient had economic difficulties, and the frequency was recorded as 1, both income and salary belonged to the same first label: income.

#### Calculation of correlation between labels

We used the corr code of Python 3.6 (Pearson correlation coefficient) to determine the correlation coefficient as an indicator of the degree of correlation between observation data. The default was α = 0.05, with a confidence level of 95% (bilateral).

## Results

### Summary of primary labels

**Fig. 4:**
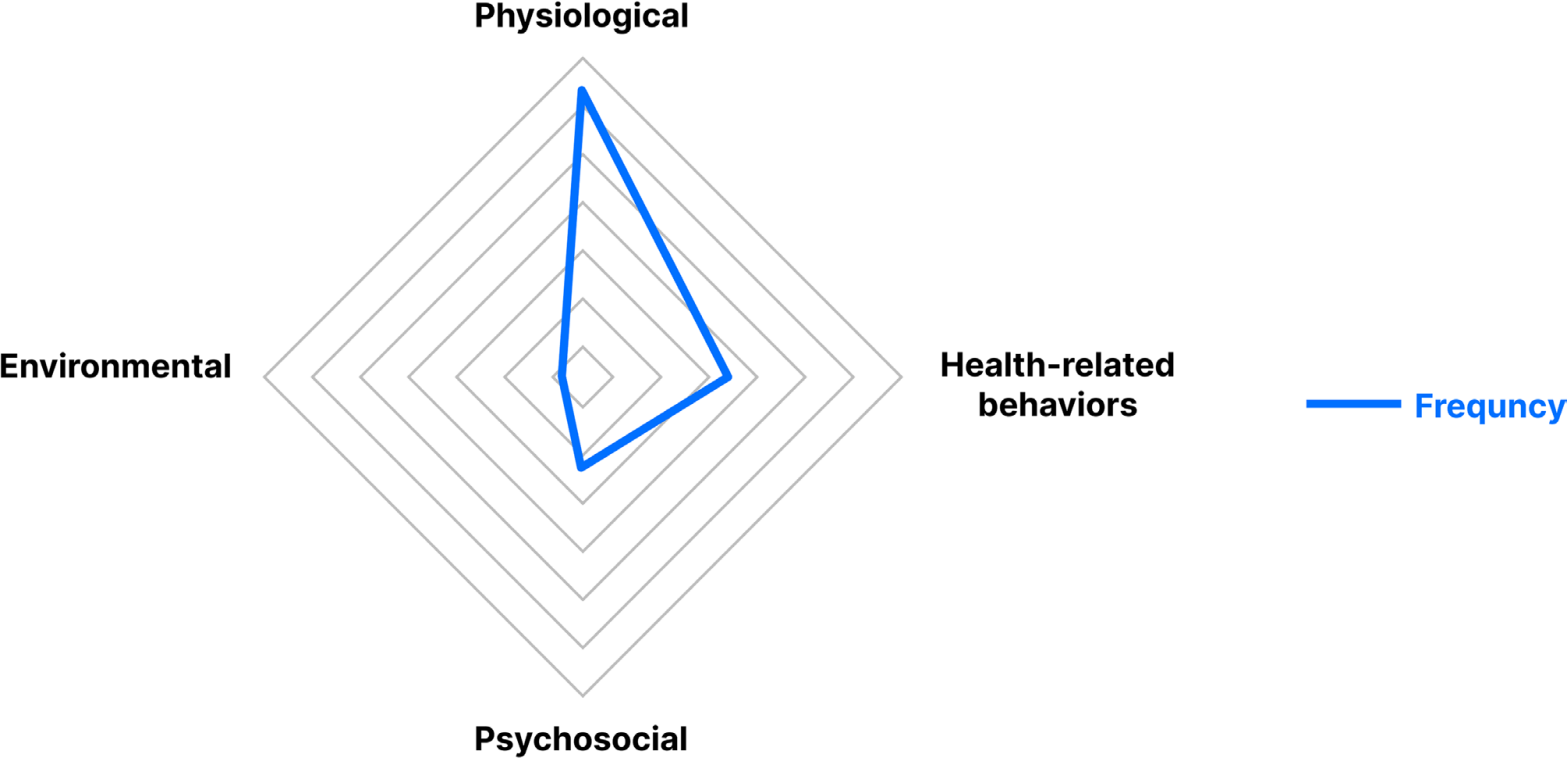
Radar chart of primary labels

Patients reported 1,246 physiological problems, 673 health-related behavior problems, 425 psychological problems, and 127 environmental problems that were identified as home-based rehabilitation problems under the primary labels. The radar chart (Fig. 4) shows that the majority of the patients’ problems were physiological.

### Summary of secondary labels

**Fig. 5:**
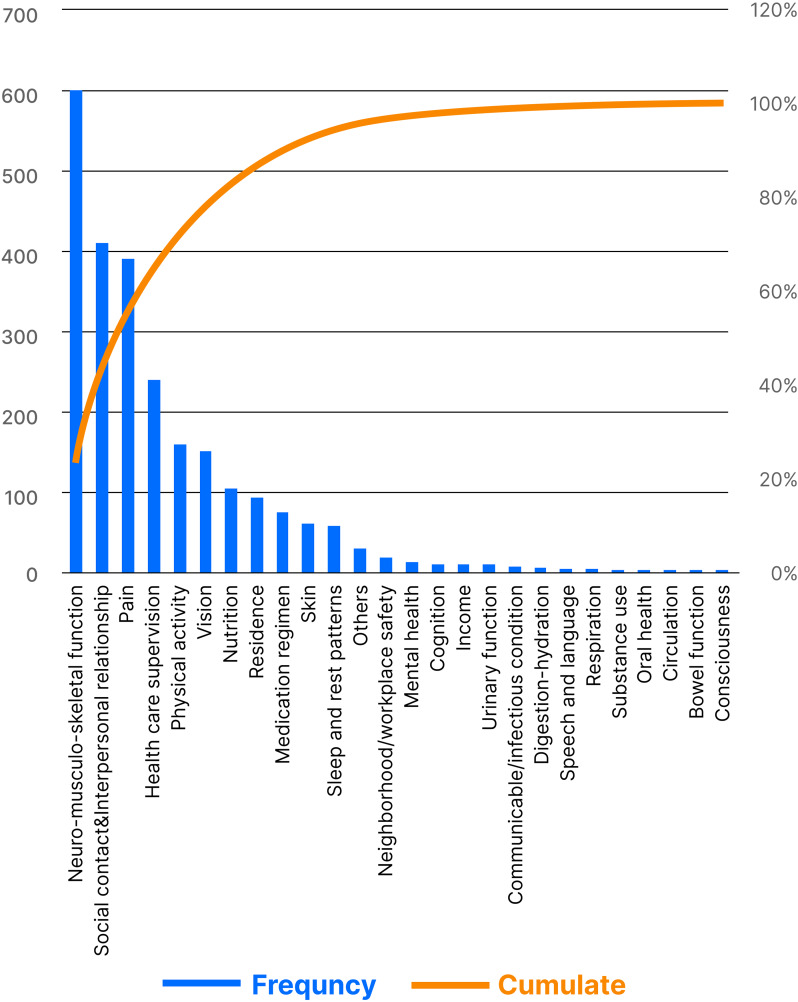
Pareto chart of secondary labels

As shown in Fig. 5, problems related to neuromusculoskeletal function, interpersonal relationships, and pain were the top three secondary labels among patients’ self-reported problems. The Pareto curve shows that more than 80% of the subjects’ health-related problems consisted of issues related to neuromusculoskeletal function, interpersonal relationship, pain, health care supervision, physical activity, vision, nutrition, and residential environment.

### Subdivision ratios of each dimension

**Fig. 6:**
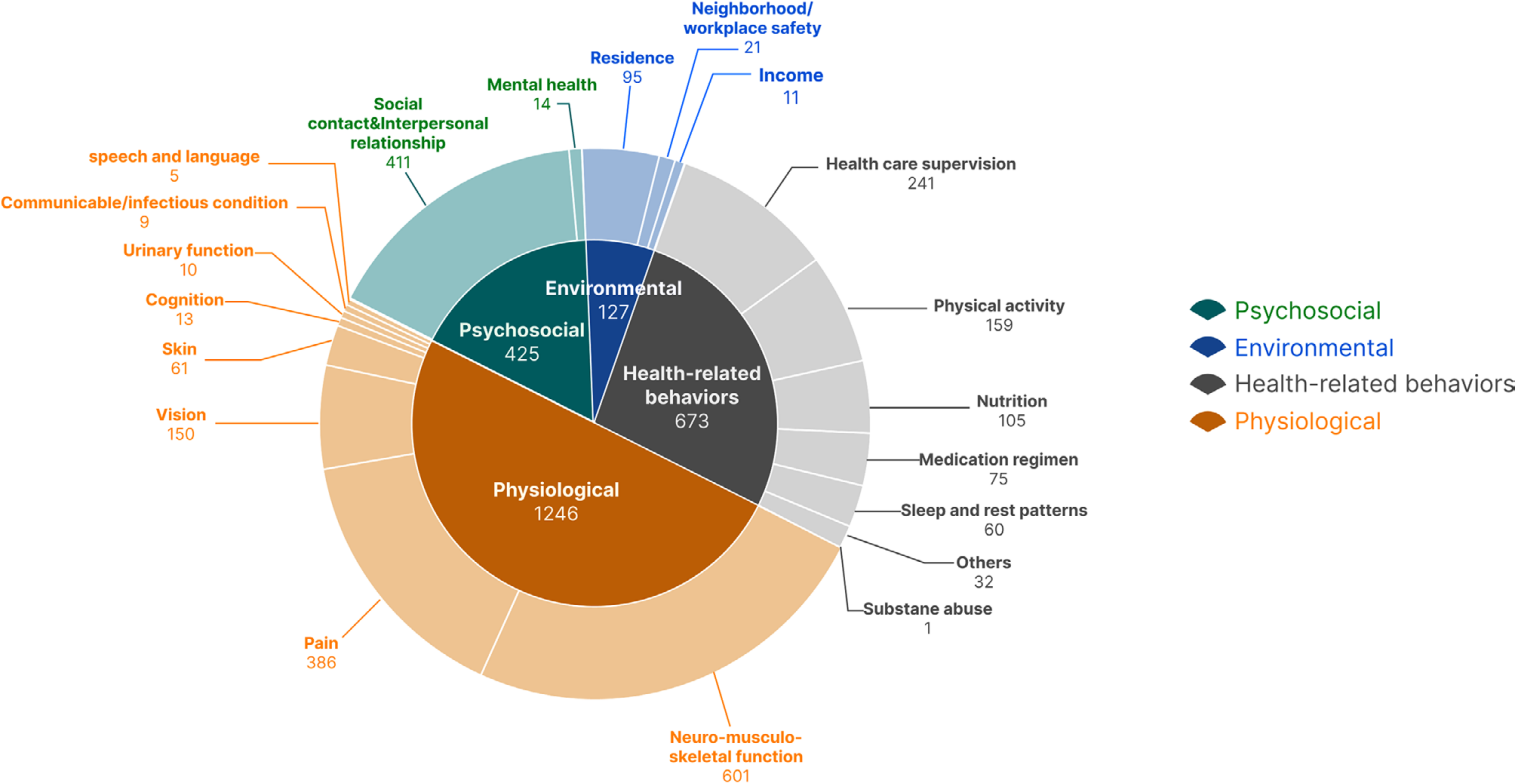
Tag summary pie charts for all labels

As shown in Fig. 6, physiology was the most frequently occurring primary label. Problems reported most by patients were related to neuromusculoskeletal function, which ranked first among the secondary labels. Interpersonal relationships was the main problem in the psycho-social dimension, and ranked second among all secondary labels. Additionally, the secondary label of health care guidance was the most frequent issue in the health-related behavior dimension. The secondary label of residential environment was the most frequent secondary label in the environment dimension.

### Frequencies of patients’ reported problems in different time periods

As shown in Table 1, the frequency of problems reported by patients at different stages of rehabilitation differed. The stage with the highest frequency of self-reported problems was 6-months postoperatively. Patients at all stages mainly reported physical problems. Compared with other stages, the frequency of psycho-social problems was higher within 1 week; physiological problems were most prominent within 1 month, and health-related behaviors were most common after 1 month and within 6 months postoperatively.

**Table 1:** Frequencies of problems reported by patients in different rehabilitation phases

Primary label	Within 1 week	Within 1 month(>1w)	2–6 months	After 6 months
Environmental	6(5.1%)	17(6.0%)	27(4.7%)	65(5.4%)
Psychosocial	24(20.3%)	51(18.1%)	81(14.1%)	210(17.4%)
Physiological	59(50.0%)	148(52.5%)	288(50.0%)	598(49.5%)
Health-related Behaviors	29(24.6%)	66(23.4%)	180(31.3%)	334(27.7%)
Total	118	282	576	1,207

Data are given as frequency (percentage of current phase)

### Correlation analysis between dimensions

This study used a non-questionnaire analysis, and deep mining of data interactions, etc. could only be performed manually on the captured data. Therefore, we used pairwise correlation analysis of all matching labels to explore the interactions between problems, and between problems and rehabilitation phases Using a significance level α = 0.05 (bilateral).and degree-of-freedom n-2 equal to 485-2, the tested statistics needed to be greater than 1.964887641 to be significant. As the primary labels were all very weakly correlated, and each label was very weakly (less than 0.2) or not correlated to the rehabilitation phases, we only considered correlations between secondary labels with r greater than or equal to 0.2.

**Table 2:** Correlation analysis between secondary labels (α = 0.05, n = 483)

Label A	Label B	Correlation coefficient	Statistics tested
Speech and language	Consciousness	0.45	10.93184132
Nutrition	Medication regimen	0.40	9.542014593
Circulation	communicable/infectious condition	0.33	7.6977035
Physical activity	Health care supervision	0.33	7.671889236
Pain	Neuromusculoskeletal function	0.29	6.709414938
Respiration	Other health-related behaviors	0.24	5.48411013
Sleep and rest patterns	Physical activity	0.23	5.222293589
Neuromusculoskeletal function	Physical activity	0.23	5.187830114
Residence	Vision	0.20	4.562193348
Sleep and rest patterns	Other health-related behaviors	0.20	4.55268952

## Discussion

### Consistency with other research

Based on our label analyses, the frequency of problems reported in the physiological dimension was the highest, particularly during the first month after surgery. Neuromusculoskeletal function issues ranked first among the secondary labels. The need for intervention in terms of neuromusculoskeletal function is a common problem after most orthopedic surgeries, and strength training can help patients achieve a better state of recovery. The actual physical function recovery of patients is reported to be very different from expectations, indicating the patient’s need for intervention [15, 16]. Nursing staff should provide functional recovery and physiological guidance. Physiological problems may also coexist with other problems, such as psychological expectations, and overall analysis is required for comprehensive judgment and intervention.

In our study, the most frequently-occurring keyword self-reported by patients from 1 to 6 months post-operatively was pain. In previous qualitative research, we found that medical staff can recognize patients’ need for pain control and provide a measure of attention. Relevant guidelines in China and other countries have reached consensus on the need for pain control after hip replacement [17]. However, as it involves medication compliance and standardization during home-based rehabilitation, many patients still do not achieve good pain control. Community hospitals and Grade A Class 3 hospitals need to establish postoperative pain medication standards. There is an increasing number of studies on compliance with home-based rehabilitation medication, but this needs to be considered specifically for older patients.

This study also analyzed the correlation between pain labels and other problems. Differences in pain related to neuromusculoskeletal function(Table 2). Chinese patients often use plasters, hot compresses, acupuncture (in hospital) and other health-related behaviors during the rehabilitation stage. Many Chinese patients refuse to take painkillers because they think they are poisonous, so the issue deserves further study.

### New problems identified

#### Chinese patients face marked problems with nutrition intervention

In this qualitative machine learning analysis patients required marked nutritional intervention (Fig. 6). In the primary label dimension of health-related behaviors, nutrition-related problems ranked third. Moreover, as shown in the secondary label correlation analysis (Table 2), nutritional problems were weakly correlated with following doctors’ medication orders (r = 0.4). The reason for the prominence of these problems in older patients in China may be related to advanced age and culture. Compared to young people, older patients will experience different degrees of decline in various physical functions, such as reduced digestive function, changes in taste, and decreased metabolic capacity [18]. There is often an imbalance between nutrient supply and consumption, causing nutrition-related problems. Moreover, in China, food is often used as medicine [19]. Patients often consult medical staff in terms of food choice during illness. Our health education for patient rehabilitation includes detailed guidance on nutrition, but only in terms of comprehensiveness and quantity from the perspective of Western medicine, while the types of food that should be selected needs to be determined jointly with medical staff. For medical staff, mastering Traditional Chinese Medicine(TCM) principles for health maintenance with food and diet therapy can provide patients with specific nutritional guidance and may be significant for medical practice.

#### The importance of the neighborhood and workplace

Neighborhood/workplace and residential environment keywords appeared 21 times and 95 times, respectively, in our analysis of related keywords. This frequency was much higher than that of infection problems, which typically concern medical staff. Previous studies showed that there is a gap between the patient’s reported improvement in the home environment and the actual situation, which requires attention [20]. Residential environment and vision are correlated to a certain degree, which suggests that the vision problems reported by the patients in this study might be caused by the residential environment. Older patients face various problems in returning to society after home rehabilitation, given that their joint functions do not yet allow use of equipment in the home, neighborhood, and workplace. Society should jointly promote construction of residential environments and workplaces that cater to the needs of older people.

#### Psychological problems urgently requiring guidance

Patients relating their real-life problems is an “open-ended” narrative. We found that patients generally had less self-reports related to psychology (Table 1). However, other “question-and-answer” studies that used questionnaires showed that older patients had a high level of related needs. Older hip replacement patients are more likely to have functional problems of the body, which affect their quality of life and leads to depression, anxiety, and other negative emotions. Depression is common in older hip fracture patients (prevalence rate of 9‒47%) [21]. Related emotions reduce the mobility of older patients, resulting in slow functional recovery. Older patients often feel inferior because they were hospitalized. In different stages of recovery, patients successively felt depression, melancholy, hopelessness, and fear for the future, which lasted for several months after discharge from the hospital, and even hampered their recovery [22]. This may be due to concerns about burdening the family, or due to Chinese culture, which is characterized by introversion and subtlety, or because patients conducting online consultation were more inclined to solve problems related to function and pain. Together, these factors led to less self-expression of negative emotions than that of other problems during open-end self-disclosure. Therefore, medical staff, particularly nursing staff closest to the patients, should guide patients in a timely manner, and actively intervene in psychological problems, thereby improving the mental health and interpersonal skills of older patients.

### The efficiency of machine learning analysis

Due to the different ways of obtaining data, the use of artificial intelligence operations to capture and use machine learning analysis is more efficient than other methods [6]. After completing the code, data were rapidly obtained in this study, while automatic data importing from traditional questionnaire surveys requires template design and entry of data in strict accordance with the specified format through a terminal. Thus, it is time-consuming and labor-intensive to collect questionnaire items on research related to home-based rehabilitation. The present study used the law of big data to obtain the maximum network data size possible, and artificial intelligence capture was also fully automated. Thus, efficiency was markedly improved compared with manual filling and data input.

### Study limitations

Limited by time and manpower, we confined ourselves to executing preliminary filtering, which was grounded in previous research and manual analysis of the corpus. This limitation necessitated the use of the keyword lexicon as the standard for classification in this study, yet it opens a pathway for future supplementation and revision to enhance precision in analysis. Consequently, the application of deep learning, specifically utilizing deep neural networks, emerges as a solution to address the challenge of feature expression. Nevertheless, the requisite of substantial training data for deep learning models presents a hurdle, especially considering the current data, mined under time and resource constraints, are yet insufficient for such methodologies. This insufficiency underscores the need for further research.

Building upon this need for further research, this study also attempted to build a correlation model between the rehabilitation phases and the patients’ self-reported outcomes. However, due to the small sample size, an accurate model could not be obtained, and only fuzzy statistics could be produced, indicating that more patient data are required to obtain an accurate model. This challenge with data sufficiency and model accuracy further amplifies the aforementioned limitations and reinforces the imperative for additional research and data collection in future studies.

Addressing another dimension, in China, many elderly individuals still do not know how to use smart devices or access the internet. Consequently, this study omits research on this particular demographic. However, as digital health product designs become increasingly age-friendly in the future, the methods employed in this study can serve as a reference for comprehensively understanding patient needs in future investigations, thereby bridging the gap between technological advancements and user accessibility, and ensuring that future studies are inclusive and representative of the entire demographic.

## Conclusions

This study explored effective methods for identifying the primary health-related issues of elderly patients during home-based rehabilitation following hip replacement surgery, through the construction of a corpus of patients’ self-reported rehabilitation care problems based on the Omaha classification system. By utilizing the Python development language and implementing artificial intelligence to match corpus data on the cooperative platform, we were able to identify and statistically analyze these issues. The results indicate that most patients primarily face physiological health-related problems, especially within the first 6 months post-surgery.

Although the method employs technology from a few years ago, it still holds significant referential value in how to rapidly investigate patient needs and acquire pertinent information. Our approach leverages existing vast data resources and, through fully automated artificial intelligence capture, significantly enhances efficiency compared to manual data entry. This provides a foundation for conducting quick, economical, and comprehensive assessments of patients using existing data resources and aids in devising appropriate patient rehabilitation care intervention strategies for the future.

Future research could further explore and optimize the application of this method, especially within larger datasets and more diverse patient populations. Moreover, with the continuous advancement of machine learning and artificial intelligence technologies, our method can also be further optimized and updated to more accurately and comprehensively identify and analyze patients’ health issues and needs during home-based rehabilitation.

Additionally, it warrants mentioning that this study was concluded prior to the implementation of the Chinese Big Data Law. Nonetheless, adherence to all pertinent regulations safeguarding patient privacy was scrupulously ensured. Looking ahead, it becomes imperative to further investigate associated issues, exploring strategies to harness data in a way that is both effective and secure, thereby fortifying clinical decision-making within the context of data generation. This underscores the paramount necessity to forge robust data management and analytic frameworks, which not only remain compliant with legal standards but also optimize the translation of data into actionable and clinically relevant insights.

### Electronic supplementary material

Below is the link to the electronic supplementary material.

Supplementary Material 1

Supplementary Material 2

Supplementary Material 3

## Data Availability

The dataset supporting the conclusions of this article is available from the corresponding author upon reasonable request.

## Abbreviations

TCM: Traditional Chinese Medicine

## References

[1] Wang HM (2019). Paying attention to the health status of the older in China and promoting the national strategy of healthy aging,Chin. J Epidemiol Chinese Journal of Epidemiology.

[2] Curtis E, Litwic A, Cooper C, Dennison (2015). Determinants of muscle and bone aging. J Cell Physiol.

[3] Li N, Liu HN, Gong XF, Zhu SW, Wu XB, He L (2016). Epidemiological analysis of hospitalized patients with femoral neck fracture in a first class hospital of Beijing. J Peking Univ Health Sci.

[4] Hoogland J, Wijnen A, Munsterman T (2019). Feasibility and patient experience of a home-based rehabilitation program driven by a tablet app and mobility monitoring for patients after a total hip arthroplasty. JMIR Mhealth Uhealth.

[5] Kingsbury SR, Dube B, Thomas CM, et al. Is a questionnaire and radiograph-based follow-up model for patients with primary hip and knee arthroplasty a viable alternative to traditional regular outpatient follow-up clinic?Bone. Joint J. 2016;98–B(2):201–8. 10.1302/0301-620X-98B2-36424. 10.1302/0301-620X.98B2.36424 26850425

[6] Ristevski B, Chen M (2018). Big Data Analytics in Medicine and Healthcare. J Integr Bioinform.

[7] Zhang L, Hall M, Bastola D (2018). Utilizing Twitter data for analysis of chemotherapy. Int J Med Inform.

[8] Le Glaz A, Haralambous Y, Kim-Dufor DH, Lenca P, Billot R, Ryan TC, Marsh J, DeVylder J, Walter M, Berrouiguet S, Lemey C (2021). Machine Learning and Natural Language Processing in Mental Health: systematic review. J Med Internet Res.

[9] Jiang T, Gradus JL, Rosellini AJ (2020). Supervised machine learning: a brief primer. Behav Ther.

[10] Martin KS, Norris J (1996). The Omaha System: a model for describing practice. Holist Nurs Pract.

[11] Altiner M, Secginli S, Kang YJ (2020). Refinement, reliability and validity of the Time Capture Tool (TimeCaT) using the Omaha System to support data capture for time motion studies. Jpn J Nurs Sci.

[12] Benke K, Benke G (2018). Artificial Intelligence and Big Data in Public Health. Int J Environ Res Public Health.

[13] Tack C (2019). Artificial intelligence and machine learning | applications in musculoskeletal physiotherapy. Musculoskelet Sci Pract.

[14] Chang Z, Lv Y (2019). Mass data cleaning system based on regular expression. J Comput Appl.

[15] Peeters CM, Visser E, Van de Ree CL, Gosens T, Den Oudsten BL, De Vries J (2016). Quality of life after hip fracture in the elderly: a systematic literature review. Injury.

[16] Gustafsson BA, Ponzer S, Heikkilä K, Ekman SL (2007). The lived body and the perioperative period in replacement Surgery: older people’s experiences. J Adv Nurs.

[17] National Guideline Centre (UK). Evidence review for long-term follow-up and monitoring: Joint replacement (primary): hip, knee and shoulder: Evidence review T. London: National Institute for Health and Care Excellence (UK); 2020 Jun. PMID: 32881449.Available from:https://www.nice.org.uk/guidance/ng157. 32881449

[18] Dong L, Xiao R, Cai C, Xu Z, Wang S, Pan L, Yuan L. Diet, lifestyle and cognitive function in old Chinese adults. Arch Gerontol Geriatr 2016 Mar-Apr;63:36–42. 10.1016/j.archger.2015.12.003 26791169

[19] Ju XR (2000). On the thought of balanced diet in Chinese medicine. Jiangsu J Trad Chin Med.

[20] Xu HL, Cheng SZ, Yan FJ (2007). Investigation and analysis of the home environment improvement needs of the older after hip fracture in Guangzhou. Mod Clin Nurs.

[21] Holmes JD, House AO (2000). Psychiatric Illness in hip fracture. Age Ageing.

[22] Liu C, Shi L, He J, Zhang YN (2020). Adverse effect of depression on surgical outcome of artificial femoral head replacement in elderly patients. Chin J Joint Surg(Electronic Edition).

