# Supplementary material for: Identifying self-reported health-related problems in home-based rehabilitation of older patients after hip replacement in China: a machine learning study based on Omaha system theory
Source: BMC Med Inform Decis Mak. 2023 Nov 21;23:268. doi: 10.1186/s12911-023-02353-7 (PMC10664483; doi:10.1186/s12911-023-02353-7)
Supplement: Supplementary file 1 — Supplementary Material 1 [file 12911_2023_2353_MOESM1_ESM.pdf]

Description of disease:

The wound seems to be recovering well after surgery, but the leg is very swollen and hurts all the time.

Condition :

Hip arthroplasty

Help desired:

How should I recover from the surgery? Is it normal to be in pain 10 days after the surgery? If so, how long will the pain last?

Length of illness:

Within one month

Medication:

Before surgery, I took Bexinol, Bering capsules, and metoprolol tartrate tablets for my heart.

History of allergy:

Allergy to painkillers prescribed by the doctor after surgery.

Previous medical history:

10 days after surgery
