# Supplementary material for: Identifying self-reported health-related problems in home-based rehabilitation of older patients after hip replacement in China: a machine learning study based on Omaha system theory
Source: BMC Med Inform Decis Mak. 2023 Nov 21;23:268. doi: 10.1186/s12911-023-02353-7 (PMC10664483; doi:10.1186/s12911-023-02353-7)
Supplement: Supplementary file 2 — Supplementary Material 2 [file 12911_2023_2353_MOESM2_ESM.pdf]

Explanatory notes for the terms in this study

Terms	Definition
machine learning	A type of artificial intelligence that enable computers to independently initiate and execute learning when exposed to new data.
supervised machine learning	A machine learning paradigm used to make predictions about future instances based on a given set of labeled paired input-output training (sample) data.
segmentation	It is a process whereby human language is converted into characters that can be recognised by machine
lexicon	Lexicon is a complementary thesaurus to the segmentation tool in specific scenarios
tag	In this study, it refers to the elements contained in each medical record text
label	A word or phrase to identify and match keywords from the text
