# Supplementary material for: Identifying self-reported health-related problems in home-based rehabilitation of older patients after hip replacement in China: a machine learning study based on Omaha system theory
Source: BMC Med Inform Decis Mak. 2023 Nov 21;23:268. doi: 10.1186/s12911-023-02353-7 (PMC10664483; doi:10.1186/s12911-023-02353-7)
Supplement: Supplementary file 3 — Supplementary Material 3 [file 12911_2023_2353_MOESM3_ESM.pdf]

EXAMPLE

As shown in figure1, we identified the text about ‘What would I like to know’(help desired) in this sample.

Description of disease.
The wound seems to be recovering well after surgery, but the leg is very swollen and hurts all the time.
Condition :
Hip arthroplasty
What I would like to know.
How should I recover from the surgery? Is it normal to be in pain 10 days after the surgery? If so, how long will the pain last?
Length of illness:
Within one month
Medication:
Before surgery, I took Bexinol, Bering capsules, and metoprolol tartrate tablets for my heart.
History of allergy:
Allergy to painkillers prescribed by the doctor after surgery.
Previous medical history:
10 days after surgery

The keyword ‘pain’ was matched with the secondary label ‘pain’ according to the lexicon, and the corresponding primary label was ‘physiological’ that shown in figure 2.

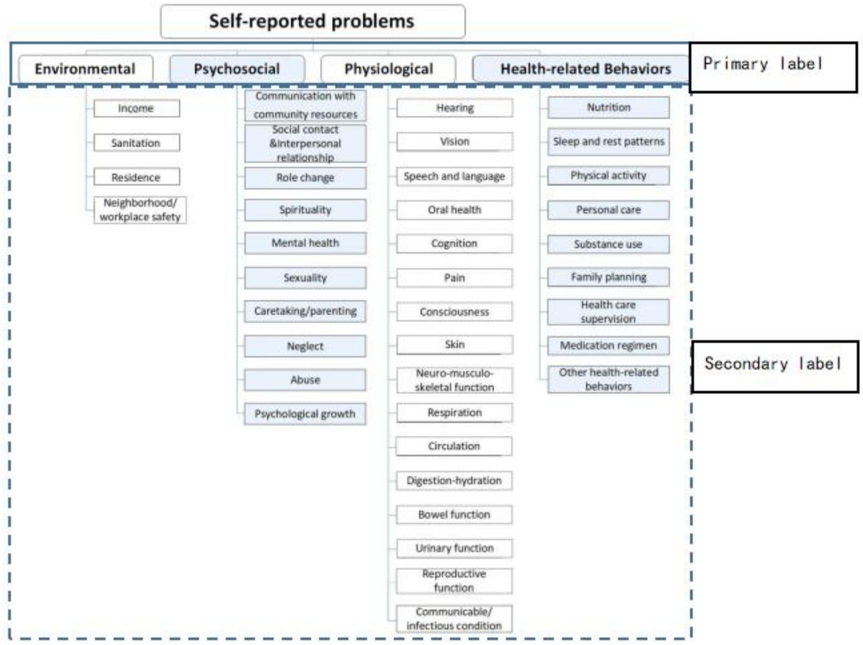

Figure 2. Classification criteria for self-reported problems in rehabilitation care after hip replacement in older patients, based on the Omaha classification system [10].

The frequency of each question in the sample was only recorded once, that is, assuming that 200 patients mentioned pain and its related words, the frequency of the secondary label ‘pain’ was been finally counted as 200.
